# Genomic and Phenotypic Characterization of a Novel Virulent Strain of *Cyvirus cyprinidallo*2 Originating from an Outbreak in The Netherlands

**DOI:** 10.3390/v17050658

**Published:** 2025-04-30

**Authors:** Bo He, Arun Sridhar, Marc Thiry, Olga Haenen, Alain F. C. Vanderplasschen, Owen Donohoe

**Affiliations:** 1 Immunology-Vaccinology, Department of Infectious and Parasitic Diseases, Fundamental and Applied Research for Animals & Health (FARAH), Faculty of Veterinary Medicine, University of Liège, B-4000 Liège, Belgium; 2 GIGA Neurosciences—Cellular and Tissue Biology, Cellular Biology, Faculty of Sciences, University of Liège, B-4000 Liège, Belgium; 3 National Reference Laboratory for Fish Diseases, Wageningen Bioveterinary Research, Wageningen University Research, P.O. Box 65, 8200 AB Lelystad, The Netherlands; 4 WEL Research Institute, Avenue Pasteur 6, B-1300 Wavre, Belgium; 5 Bioscience Research Institute, Technological University of the Shannon, N37 HD68 Athlone, Co., Westmeath, Ireland

**Keywords:** *Cyvirus cyprinidallo*2, *Cyprinid herpesvirus* 2, CyHV-2, cyprinivirus, *Alloherpesviridae*, *Carassius auratus*, aquaculture

## Abstract

*Cyvirus cyprinidallo*2 (CyHV-2) is the causative agent of herpesviral hematopoietic necrosis in several economically important farmed freshwater fish species of the genus *Carassius*. Despite several CyHV-2 strains being isolated and fully sequenced, there is a lack of detailed characterization and consistent information on strains that exhibit high virulence in adult goldfish through viral challenge by immersion, particularly in the context of European strains and host populations. Strains that can cause highly virulent disease via this inoculation route are much more compatible with experimental designs that are representative of natural infection; thus, their utilization provides greater biological relevance. Consequently, in this study, we isolated three novel strains of CyHV-2 (designated NL-1, NL-2, and NL-3), originating from outbreaks in The Netherlands. Full-length genome sequencing and phylogenetic analyses revealed that these newly isolated strains are distinct from known strains and from each other. Significant differences were observed between the strains, in terms of in vitro growth kinetics, with NL-2 exhibiting stable passaging and superior fitness in vitro. Importantly, the challenge of adult Shubunkin goldfish with the NL-2 strain via immersion (2000 PFU/mL) induced an average mortality of ~40%, while parallel experiments with the CyHV-2 reference strain ST-J1 resulted in no mortality. Taken together, this study represents the characterization of a new CyHV-2 in vivo infection model, much more compatible with experimental designs that are required to be representative of natural infection. This model will be extremely useful in many aspects of CyHV-2 research in the future. Importantly, the genetic and phenotypic characterization performed in this study generates hypotheses on the potential roles of CyHV-2 genes in adaptation of the virus in vitro or in vivo.

## 1. Introduction

Among the viruses that infect widely cultivated freshwater fish, viral species from the family *Alloherpesviridae* can be particularly problematic. Disease caused by these viruses can lead to high mortality and large economic loss within the freshwater aquaculture industry [1,2], which is an extremely important sector in the context of global food security [3]. Within the family *Alloherpesviridae*, viruses belonging to the recently renamed genus *Cyvirus* [4] (formerly referred to as *Cyprinivirus* and collectively referred to as cypriniviruses [5,6]), can be among the most pathogenic, leading to high mortality outbreaks when water temperatures are optimum for viral replication [1,2]. Some of these cyvirus species are relatively well studied, including *Cyvirus cyprinidallo*2 [4] (or CyHV-2, formerly known as *Cyprinid herpesvirus 2*). CyHV-2 is the causative agent of a disease referred to as herpesviral hematopoietic necrosis disease (HVHND), which is associated with elevated mortality rates in goldfish (*Carassius auratus*) and related species such as crucian carp (*Carassius carassius*) and gibel carp (*Carassius gibelio*), as well as other *Carassius* spp. [7,8]. The disease caused by CyHV-2 was initially documented in 1995, and since then, its global prevalence has increased substantially [9,10]. It represents a significant threat to the sustainability of goldfish aquaculture and the culture of other economically important species such as gibel carp and crucian carp, which are farmed for consumption and thus represent an important food production sector [10].

As per other members of the genus *Cyvirus* [11,12], live-attenuated vaccines against CyHV-2 have been demonstrated to be effective [13,14]. Notably, previous CyHV-2 vaccine development studies involving goldfish, utilized the Wakin-variety goldfish as models, with viral challenge trials conducted via immersion in water contaminated with CyHV-2 [13]. However, recently, we observed notable resistance to CyHV-2 challenge by immersion among adult goldfish populations sourced from European suppliers, including the popular Shubunkin goldfish breed. This was observed with the CyHV-2 ST-J1 strain, which is the designated CyHV-2 reference strain in GenBank (NC_019495.1). Notably, this was despite the fact that this strain was initially isolated from goldfish undergoing clinical disease [7]. We also observed a similar outcome using the CyHV-2 YC-01 strain using the same populations [15].

The Shubunkin goldfish breed is heavily associated with international trade [16] and thus represents a highly epidemiologically and economically relevant model in terms of vaccine development. Furthermore, Shubunkin goldfish are relatively easy to maintain and reproduce, which is extremely useful in terms of sustaining a supply of hosts for experimental infection trials. However, the lack of a broadly virulent and lethal CyHV-2 strain that can be used with this model, particularly when long-term, stable culture in vitro and infection by immersion in vivo is required, limits the study of CyHV-2 pathogenesis in the most biologically relevant contexts.

Despite the clear resistance to some CyHV-2 strains (including the ST-J1 reference strain) exhibited by some goldfish breeds (or at least some populations thereof), novel uncharacterized or recently emerged strains of CyHV-2 still remain a problem for both domesticated and wild *Carassius* spp. [16,17]. Consequently, in the present study, we sought to identify novel CyHV-2 strains from ongoing outbreaks that were (i) compatible with long-term stable culture in vitro and (ii) capable of causing sufficient and consistent virulence and lethality in Shubunkin goldfish when challenged by immersion. If successful, this would facilitate the establishment of a stronger CyHV-2 in vivo infection model to use in advancing our understanding of this virus and the development of effective disease mitigation strategies.

As part of this process, we successfully isolated and cultured three novel CyHV-2 isolates from different internal organ samples originating from CyHV-2 outbreaks among goldfish and gibel carp in The Netherlands, which we designated NL-1, NL-2, and NL-3. We then compared their genetic characteristics to those of other sequenced strains. Subsequently, in order to identify the optimum strain to use as part of a future CyHV-2 in vivo infection model, we compared their biological properties in vitro and in vivo. Through this process, we selected the most suitable isolate for further comparison to the CyHV-2 ST-J1 reference strain. This revealed that the NL-2 isolate was (*i*) highly compatible with long-term stable culture in vitro and (*ii*) much more virulent in a CyHV-2 Shubunkin goldfish model compared to the CyHV-2 ST-J1 reference strain. Importantly, in contrast to the reference strain, the NL-2 strain was able to induce mortality after inoculation by immersion in infectious water. These properties make the NL-2 isolate more valuable for incorporation into CyHV-2 Shubunkin goldfish studies. Finally, the full-length genome sequencing of the new isolates generated data on CyHV-2 genetic diversity and created new hypothesis for the identification of key CyHV-2 virulence factors.

## 2. Materials and Methods

### 2.1. Sample Collection and Handling

Three independent pools of internal organs (spleen, heart, kidney, pooled from up to ten fish per pool) were collected between 2016 and 2018 in The Netherlands from diseased goldfish and gibel carp. Samples were designated by the names NL-1 (unknown goldfish breed; WBVR 16009271), NL-2 (Shubunkin goldfish; WUR-NL-18015159-3), and NL-3 (wild gibel carp; WUR-NL-16010689). Each sample was processed separately. Samples were first homogenized with pestle and mortar and resuspended in 2 mL of sterile M199 medium (HEPES buffered; Sigma, Kawasaki, Japan); the suspension was clarified by centrifugation at 3000× *g*, 4 °C for 15 min (Allegra X-15R, Beckman, Brea, CA, USA). After centrifugation, the supernatant was aseptically separated from the tissue pellet. The supernatant was filtered twice using a sterile cell strainer with a diameter of 100 µm, then filtered with a sterile disposable syringe filter with a pore size of 0.22 µm. Purified supernatant was then aliquoted and stored at −80 °C (designated as passage #0 or p#0). DNA extraction was performed with the remaining tissue pellet using a commercial nucleic acid extraction kit (QIAamp DNA Mini Kit, QIAGEN, Hilden, Germany). DNA samples were stored at −20 °C for further analysis.

### 2.2. In Vitro Methods

#### 2.2.1. CyHV-2 PCR Detection

To confirm the presence of CyHV-2 in the samples, the DNA extracted from the tissue pellets was analyzed by PCR. Briefly, this was performed using the High-Fidelity PCR Mix Buffer (New England Biolabs, Ipswich, MA, USA) per manufacturer instructions, with a total reaction volume of 25 µL and 2 µL template. The forward and revers primers were 5′-GGACTTGCGAAGAGTTTGATTTCTAC-3′, and 5′-CCATAGTCACCATCGTCTCATC-3′, respectively (as described by [18]). The PCR cycling conditions included an initial denaturation at 98 °C for 2 min, followed by 35 cycles at 98 °C for 30 s, 60 °C for 45 s, 72 °C for 45 s, and a final extension at 72 °C for 10 min. The PCR products were loaded into electrophoresis gels and imaged using an ImageQuant 800 CCD imager (Cytiva, Marlborough, MA, USA).

#### 2.2.2. Cells and Virus

The RyuF-2 cell line kindly provided by Prof. Sano (Tokyo University of Marine Science and Technology, Tokyo, Japan) was used to propagate all viral isolates. Cells were cultured at 25 °C using Medium 199 (HEPES buffered; Sigma, Kawasaki, Japan), supplemented with 10% fetal bovine serum (FBS; Gibco, Life Technologies, Carlsbad, CA, USA), penicillin (100 U/mL), streptomycin (100 μg/mL), and Amphotericin B (0.25 μg/mL). The CyHV-2 ST-J1 strain (NC_019495.1) was used as the reference strain in this study. All isolates were cultured in RyuF-2 cells at 25 °C with no CO_2_ using the cell culture media described above, supplemented with goldfish kidney extract (final concentration 0.2 *v*/*v*%, prepared as Shibata et al. described utilizing Shubunkin goldfish kidney) [19].

#### 2.2.3. Initial In Vitro Culture and Characterization of the CyHV-2 Isolates

Serial dilutions (10-fold) of the purified organ supernatant (NL-1 #0; NL-2 #0, and NL-3 #0) were prepared. Briefly, 100 µL of supernatant was mixed with 900 µL serum-free sterile medium, and this was repeated to create a dilution series ranging from 10^−1^ to 10^−3^. Six-well plates with RyuF-2 fresh cells (100% confluent, approximately 24 h post-seeding) were inoculated with 800 µL of diluted supernatant per well, and the cells were incubated for 2 h at 25 °C. After this initial incubation, the initial inoculum was removed from each well and replaced with 2 mL sterile complete cell culture medium (0.2% kidney extract added) containing 2% *w*/*v* carboxymethylcellulose (CMC) [19]. These plates were then incubated for 10 days at 25 °C. Cytopathic effect (CPE) was visually inspected and imaged using an optical microscope (Olympus CKX41, Tokyo, Japan), followed by indirect immunofluorescence staining as described previously [15]. The stained samples were imaged using an epifluorescence microscope (Leica DM2000, Leica Microsystems, Wetzlar, Germany).

#### 2.2.4. Plaque Purification and Amplification

The earlier experimental procedure describing the initial in vitro culture and characterization of the isolates was replicated. At 10 dpi (days post-inoculation), using an optical microscope, isolated viral plaques were identified in inoculated wells. Infectious material was collected by submerging a sterile pipette tip in the 2% CMC complete media, allowing it to come into physical contact with the center of the plaque, and aspirating 10 μL of the culture medium. This 10 μL media was then transferred to 90 μL of sterile serum-free culture medium, as a means of collecting plaque-purified infectious material for later use. These 100 μL samples of plaque-purified isolates were immediately stored at −80 °C. Three independent viral plaques were purified for each isolate and designated as the first passage #1 (or p#1). In order to amplify these plaque-purified sub-cultures, they were serially passaged in cell culture flasks. As part of this process, the 100 μL of each isolate was thawed, diluted in 900 μL sterile complete medium, and used to inoculate confluent cell culture flasks (175 cm^2^). After two hours, kidney extract was aseptically added to the culture medium to reach a final concentration of 0.2%. Once CPE became widespread, viral culture medium was collected and centrifuged at 300× *g* for 5 min at 4 °C, and the viral supernatant was split into 1 mL aliquots. These aliquots were either immediately stored at −80 °C or used to inoculate new cell culture flasks as part of serial viral passaging, with viral culture proceeding as described above.

#### 2.2.5. Viral DNA Extraction and Restriction Fragment Length Polymorphism (RFLP) Analysis

Plaque-purified sub-cultures were diluted in 1 mL complete culture medium (NL-1 #1, NL-2 #1, and NL-3 #1) and used to inoculate confluent cell culture flasks (175 cm^2^), as described earlier. At 10 dpi, viral culture medium was collected and centrifuged at 3000× *g*, 4 °C for 15 min (Allegra X-15R Centrifuge, Beckman, Brea, CA, USA) to remove cell debris. Viral supernatant (35 mL from each 175 cm^2^ flask) was transferred to ultracentrifuge tubes and centrifuged at 100,000× *g* for 1 h at 4 °C. The supernatant was discarded after centrifugation, and residual liquid drops on the tube borders were removed with a swab. The viral pellet was suspended in 1 mL sterile TE buffer (0.1% NP40) and incubated in a 37 °C water bath for 20 min. It was then incubated at 56 °C for an additional 10 min to accelerate pellet dissolution. 30 mL of cold TE buffer (4 °C) was added to the tube after complete pellet dissolution. Then, 5 mL of cold 30% sucrose was added to the bottom of the tube using a glass Pasteur pipette. The tube was centrifuged at 100,000× *g* for 2 h at 4 °C. After this, the supernatant was discarded, and the pellet was resuspended in 450 μL of TE buffer. A total of 50 μL of filtered 10% SDS and 20 μL of proteinase K (25 mg/mL) was added and mixed thoroughly. The tube was covered with a lid and incubated at 56 °C in a water bath for 2 h, shaking occasionally. After this, the sample was transferred to a 2 mL tube and mixed with 510 μL phenol chloroform (4 °C). The mixture was agitated and then centrifuged at 18,000× *g* for 15 min at 20 °C. After centrifugation, the aqueous phase was carefully removed and transferred to a new 2 mL tube, and the volume was topped up to 500 μL with ddH_2_O. Then, 1 mL of absolute ethanol and 50 μL of 3M sodium acetate were added to the mixture. The sample was submerged in liquid nitrogen until frozen, then thawed by warming in a 37 °C water bath until it formed a jelly-like consistency. At this point, it was centrifuged at 18,000× *g* for 15 min at 4 °C. The supernatant was discarded, and 600 μL of 70% ethanol was added, followed by centrifugation again at 18,000× *g* for 15 min at 4 °C. The pellet was air-dried at room temperature (RT) until it became transparent. It was then resuspended in 50 μL ddH_2_O by incubating overnight at 4 °C. After this, the extracted and purified viral DNA was aliquoted and stored at −20 °C for future use. The genomic diversity of the isolated strains was then investigated by SacI restriction RFLP analysis. Briefly, 3 µg of genomic DNA was digested using SacI (New England Biolabs, Ipswich, MA, USA). After 6 h of digestion, fully digested DNA productions were separated in 0.8% electrophoresis agarose gel at 60 V for 18 h and imaged.

#### 2.2.6. Genome Sequencing, Assembly, Phylogenetic and Mutation Analysis

DNA was extracted from 3 independent plaque-purified subcultures from NL-1, NL-2, and NL-3 (9 samples in total) as described earlier, and full-length genome sequencing was performed. DNA samples were submitted for Illumina paired-end sequencing (MiSeq Kit v3 600 cycles). Raw reads (in fastq format) were processed using BBduk (v38.26) [20] facilitating adaptor sequence removal and quality trimming. This was followed by an assessment of processed fastq files using FastQC (v0.11.8). Processed reads were used as input for reference guided assembly using an in-house modified version of the popular de Bruijn graph-based assembly tool “spades” v3.15.2 [21].

The modifications to the publicly available version of spades specifically involved increasing the maximum kmer size from a default of 127 to 251, thus utilizing larger portions of individual reads during the assembly process, with the aim of improving the performance of spades, specifically the assembly of low-complexity regions, where ambiguous assembly (regarding the number of repeat units) reduced assembly scaffold contiguity. To achieve this, the following modifications were made to the publicly available source code: (*i*) Line 224 in the spades_compile.sh script was replaced with “cmake -G “Unix Makefiles” -DCMAKE_INSTALL_PREFIX = “$PREFIX” -DSPADES_MAX_K = 251 $* “$BASEDIR/src”, (*ii*) CMake (v3.15.3) was loaded in local Linux environment, and the modified compilation script was run per standard installation instructions. (*iii*) After compilation and the creation of the python script “options_storage.py” in /share/spades/spades_pipeline/, this new script was edited at line 59 to “MAX_K = 251” to ensure longer kmers would be tolerated when running spades, and line 74 was changed to “K_MERS_250 = [21, 33, 55, 77, 99, 127, 249]” to ensure that longer kmers were automatically included when spades generated combined assembly from different kmer sizes (note: for memory reasons, a value of 249 is used here as it is just under the new maximum limit of 251). (*iv*) As these modifications to utilize longer kmers caused run failure in initial testing due to memory issues, lines 69 and 70 of the python script “options_storage.py” were also changed to “THREADS = 32” and “MEMORY = 800” respectively, thus allowing assembly processes to successfully run to completion. The assembly quality was assessed via QUAST [22] (v5.2.0), which revealed improved N50 metrics (measurement of contiguity of assemblies) using the in-house version of the spades tool compared to the publicly available version.

Assembly graphs were generated using the in-house modified version of spades. The resulting scaffold(s), in .gfa format, were opened up using Bandage (v0.8.1) [23] for inspection and alignment to a local BLAST database representing the CyHV-2 reference strain, ST-J1 (NC_019495.1). Individual scaffolds mapping to reference sequences, and thus collectively representing the full-length, assembled genome, were identified from inspection of the BLAST output in Bandage. Where multiple scaffolds matched the reference genome, full-length genomes were derived from concatenating these scaffolds based on overlaps at their terminals, which was performed using SnapGene (v6.2.1) [24]. For isolates NL-1, NL-2, and NL-3, there were three reassembled genomes (each representing an independent plaque-purified subculture). Taking each isolate separately, genome reassemblies of the three plaque-purified subcultures were aligned to each other using MAFFT (v7) [25], and the output alignment was exported as a fasta file. This revealed little or no difference between plaque-purified subcultures derived from the same isolates. The alignments of genomes from these subcultures were imported into SnapGene to generate consensus sequences for each isolate (>50% agreement).

Once the consensus sequences for NL-1, NL-2, and NL-3 were established as described earlier, these three sequences were aligned to other complete CyHV-2 genome sequences. Complete CyHV-2 genome sequences for this analysis were compiled based on the accession numbers of fully sequenced CyHV-2 genomes listed in the NCBI Viral Genome Browser [26,27]. This consisted of fully sequenced genomes from the following CyHV-2 strains: CNDF-TB2015 (MN201961.1), YZ-01 (MK260012.1), SY (KT387800.1), SY-C1 (KM200722.1), ST-J1 (NC_019495.1), and YC-01 Unverified (MN593216.1). This analysis also included an additional full length sequence of the YC-01 isolate that was also sequenced and assembled in-house as described earlier (this specific YC-01 isolate was available to us from a previous study we conducted [15]). These full-length CyHV-2 genomes were aligned to the CyHV-2 ST-J1 strain using MAFFT, and the resulting alignment was exported as a fasta file and then imported into MEGA (v11) [28] as an alignment. This alignment was then saved as a MEGA format data file (.meg). This MEGA format file was opened up in MEGA and subsequently used to generate a phylogenetic tree using the UPGMA method.

In order to understand the consequences of the differences between the newly assembled genomes and the CyHV-2 ST-J1 reference genome, a list of differences relative to the reference genome were compiled. This was followed by inspection of their genomic context (i.e., coding/noncoding region etc.) and consequences (synonymous, non-synonymous amino acid change, frameshifts, ORFs impacted etc.). To achieve this, the consensus sequence for each fully assembled isolate was aligned to the CyHV-2 ST-J1 strain using MAFFT, and the resulting alignment was exported as a fasta file. All differences in the consensus relative to ST-J1 were summarized in variant call format (VCF) by converting the fasta alignment to a VCF file using the msa2vcf.jar tool [29,30]. The VCF file was opened in Excel, and each variant coordinate, which was in the context of the alignment only, was transformed to the corresponding coordinates in the ST-J1 reference strain. This was achieved by using an Excel formula to take into account the cumulative gaps in the ST-J1 and consensus sequence that had occurred prior to each variant call. The updated coordinates of these variants were verified by manually inspecting the raw read mapping data. This was done by building an index of the ST-J1 reference sequence and mapping processed reads to from each new isolate to the index using HISAT2 (v2.1.0) [31,32]. The mapping output was then converted from SAM to compressed BAM format using SAMTools (v1.9) [33], and finally, raw mapping data (BAM format) was inspected using Integrative Genomics Viewer (IGV) (v2.8.0) [34]. Inspection involved sampling random variants described in the updated VCF file (coordinates and expected differences relative to ST-J1 reference) and using IGV to locate and verify the presence of these differences in reads from new isolates that were mapped to the ST-J1 reference by HISAT2.

The updated VCF files (with variant coordinates corresponding to the ST-J1 reference) were utilized to identify the consequence of each mutation in the consensus sequence relative to the ST-J1 reference strain using VEP (v107.0) [35]. To achieve this, the updated VCF, the ST-J1 sequence (fasta format) and ST-J1 feature coordinates (GFF format) were used as input for VEP. Using a combination of grep, sort and bgzip tools in Linux, the first line of the GFF file was removed, contents were sorted by coordinates, and finally, it was compressed in .gz format before being used as input for VEP.

Maps of each fully assembled genome were generated using Geneious Prime (v11.0.4+11) [36]. First, using SnapGene, the sequence of each assembled genome was annotated with the location of CyHV-2 ORFs and terminal repeats by importing features (≥99% similarity) from a separate SnapGene file of the ST-J1 genome (initially downloaded as .gb file from GenBank, opened in SnapGene and saved as SnapGene format .dna file). Using SnapGene, the annotated versions of each newly assembled genome were saved as GenBank (.gb) files and imported into Geneious Prime. These maps were converted into GFF files and exported from Geneious Prime in GFF format. These GFF files were opened in Excel and modified to remove excess annotation. The fasta files for each assembly were then imported into Geneious Prime, along with the modified GFF files defining ORF annotations, and together, they were used to generate a map of each fully assembled genome with ORFs. The coordinates of all sequence variants relative to the ST-J1 reference strain were also added these maps by importing another GFF file defining the locations of each variant. This GFF file was generated by converting VCF files (in this case, the earlier VCF files used as input for VEP) into GFF format using the python script vcf_gff.py [37,38]. After this, the GFF was opened in Excel, and the locations of each variant (initially provided as ST-J1 coordinates) were converted to their corresponding coordinates in each newly assembled isolate using an Excel formula (described earlier). The lengths of all variants in the GFF file were calculated by utilizing the variant sequence information, which was stored in the “attributes” field (column 9) as a consequence of earlier conversion from a VCF file. Using this length information, Excel formulas were used to classify each variant as either a single nucleotide variant (SNV) or multiple-base-change/indel. This information was added to the “type” field (column 3) for later utilization by Geneious Prime when generating maps. Feature names were also deleted from the “attributes” field of the GFF using Excel. This modified GFF was then imported into Geneious Prime and used to update the maps with the location of each variant relative to the ST-J1 strain. Maps were then exported as PNG images.

All Linux-based work on this project was conducted by our team using NIC5 computing cluster, which is part of the Consortium des Équipements de Calcul Intensif (CECI) and maintained by ULiège.

#### 2.2.7. Viral Growth Assay

Viral growth assays were conducted using the same inoculation method. CyHV-2 supernatant was diluted in serum-free media to achieve a specific multiplicity of infection (MOI) in each well. Triplicate cultures of RyuF-2 cells grown in 6-well plates were infected with 1 mL viral supernatant. After a 2 h incubation period, the cells were washed with sterile, serum-free media and overlaid with complete, sterile cell culture medium supplemented with 0.2% kidney extract. At selected timepoints post-infection, both viral supernatant and infected cells were collected from each well, and the mixture was separated by centrifugation and stored at −80 °C. These samples were later thawed and analyzed by viral titration using triplicate plaque assays in RyuF-2 cells. Different inoculum doses and sampling time points were used across various experiments (*i*) An MOI = 0.0001 was used for the comparison between NL-1 #10, NL-2 #10, and NL-2 #2, with time points at 8, 12, and 16 dpi. (*ii*) An MOI = 0.01 was used for the comparison between ST-J1 #10 and NL-2 #10, with time points at 4, 6, 8, and 10 dpi.

#### 2.2.8. Viral Plaque Size Assay

RyuF-2 cells were cultured in six-well plates and inoculated with 100 PFU/well of virus, followed by a 2 h incubation period. After incubation, the cells were washed with serum-free medium and then overlaid with culture medium supplemented with 1% (*w*/*v*) CMC and 0.2% kidney extract. At different time points, viral plaques were visualized by indirect immunofluorescent staining as described in the CPE Imaging section. After a final wash with PBS, 20 randomly selected individual plaques were imaged using the Incucyte live cell analysis system (Sartorius, Goettingen, Germany), and their areas were measured manually using ImageJ software (Version 2.14).

### 2.3. In Vivo

#### 2.3.1. Fish

Two different sizes of Shubunkin goldfish (*Carassius auratus*) were utilized in this study, both at the adult developmental stage. The larger fish had an average weight of 12 g (12 ± 1.4 g), while the smaller fish group averaged 6 g per fish (5.84 ± 0.6 g). Live, mature Shubunkin goldfish were obtained from an accredited commercial company (Ruinemans Aquarium, Montfoort, The Netherlands). Microbiological, parasitic, and clinical examinations were conducted immediately upon arrival at the animal facility and then monthly to monitor for fish health. PCR analysis of a kidney homogenate confirmed that the adult goldfish from the colony were free of CyHV-2. Fish were maintained in 60 L freshwater recirculation tanks at 25 °C until they were transferred to A2 facilities for infection experiments.

#### 2.3.2. Infection of Fish with CyHV-2

Different modes of inoculation were used depending on the different test subjects and viral strains. (*i*) Basic evaluation of the inherent pathogenicity of NL-1, NL-2, and NL-3: The original supernatant (p#0) of the three new isolates (NL-1 #0, NL-2 #0, and NL-3 #0) was diluted 10-fold using serum-free medium and kept on ice. For this preliminary and exploratory experiment, due to the low amounts of p#0 material available, the viral titers in NL-1 #0, NL-2 #0, and NL-3 #0 were not established prior to use. However, viral titers were established, and viral doses were normalized, for later experiments (see later). Shubunkin goldfish (big: average weight 12 ± 1.4 g; small: 5.84 ± 0.6 g) were anesthetized by immersion in water containing benzocaine (25 mg/L). Once the fish were anesthetized, the virus was injected intraperitoneally using a 300 µL syringe (BD Micro-Fine), with each fish receiving a volume of 100 µL (big) or 50 µL (small). After injection, the fish were placed in an aerated recovery bath for 10 min and then returned to the tank. Similarly, a mock group was injected with the same dose of sterile culture medium. The experiment included 10 fish per group. Survival status and clinical manifestations were recorded daily, and the survival rate was calculated at the end of the experiment. At 5 dpi, 10 sentinel goldfish were added to each tank for cohabitation experiments. (*ii*) Comparison of in vivo pathogenicity of NL-2 strain and ST-J1 reference strain after 10 in vitro passages: The concentrations of NL-2 #10 and ST-J1 #10 were adjusted using culture medium to a final concentration of 2 × 10^4^ PFU/mL. After the administration of anesthesia as described above, inoculation was performed by intraperitoneal injection. Smaller Shubunkin goldfish (5.84 ± 0.6 g) were used, with three replicates of 10 fish for each virus strain. Each fish received a dose of 1000 PFU in 50 µL. The mock group was treated the same way, except sterile complete culture medium was injected instead of virus. Survival status and clinical manifestations were recorded daily, and the survival rate was calculated after 30 days post-injection. At 5 dpi, two replicate groups for each virus strain were selected and placed with the same number of sentinel goldfish, and fish status was monitored as described earlier for 30 days. (*iii*) Comparison of virus pathogenicity after inoculation by immersion after 10 passages: The fish (5.84 ± 0.6 g) were inoculated by immersion in water containing the virus, thus simulating the natural infection route. ST-J1 #10 and NL-2 #10 were diluted ten-fold using water at 25 °C to reach a final concentration of 2000 PFU/mL. After a 2 h immersion (10 fish/L), the fish were returned to the 60 L tank. The mock group underwent the same process using sterile complete viral culture medium. Three replicates were included for each virus strain, with 10 fish in each replicate. Survival status and clinical symptoms were recorded daily, and the survival rate was calculated after 30 days post-injection.

#### 2.3.3. Transmission Electron Microscopy

Kidney tissue was sampled from a moribund goldfish which was inoculated by IP injection using 100 μL of NL-2 (p#0, 10-fold dilution). It was fixed in 2.5% glutaraldehyde in 0.1 M Sörensen’s buffer (pH 7.4) for 2 h at room temperature. After several washes in the same buffer, the samples were post-fixed for 60 min with 2% osmium tetroxide in Sörensen’s buffer, washed in the same buffer, dehydrated at room temperature through a graded ethanol series (70, 90, and 100%), and embedded in Epon for 72 h at 60 °C. Ultrathin sections (70 nm thick) were cut using an ultramicrotome (Ultracut S, Leica, Wetzlar, Germany) equipped with a diamond knife (Diatome, Sierre, Switzerland), mounted on copper grids, contrasted in the dark for 15 min in uranyl acetate solution and for 15 min in lead citrate solution, and examined under a Jeol TEM JEM-1400 transmission electron microscope at 80 kV. Random fields were photographed using an 11-megapixel camera system (Quemesa, Olympus, Tokyo, Japan).

### 2.4. Statistical Analysis

Two-way omnibus tests on data from the viral growth curve and plaque size experiments were conducted. First, a two-way ANOVA model was generated using the anova function in R (v4.2.2) (part of the R core package) [39]. The residuals of this model were checked for normality using the Shapiro–Wilk test, implemented using the shapiro.test function in R (part of the R core package). If normal distribution was observed, the significance of each variable on outcome was taken from the two-way ANOVA model, and multiple comparisons were conducted using a Tukey post hoc test, implemented using the TukeyHSD function in R (part of the R core package). In the cases where normal distribution was not observed, analysis was performed using a generalized linear model (GLM) implemented using the glm function in R (part of the R core package), with Family option: “gamma” and Link option: “log”. To determine the significance of each variable on outcome, the model was analyzed using type III sum of squares test, implemented using the Anova tool from the R “car” package (v3.0-6) in R [40], with the default contrast coding in R adjusted appropriately prior to the use of the glm function to facilitate the use of type III sum of squares test. In such cases, post hoc multiple comparison tests were conducted on the same model using a least squares means test, implemented using lsmeans tool from the “lsmeans” package (v2.3.0) in R [41], with BH correction. Survival curves were compared using the log-rank test, implemented in GraphPad prism 8.0. All graphs were also generated using GraphPad 8.0. Only significant *p*-values (<0.05) are reported in Section 3. For the purposes of visual clarity, only results from post hoc multiple comparisons are indicated in each corresponding figure and are represented using the following symbols: ns = not significant, * = *p* < 0.05; ** = *p* < 0.01; *** = *p* < 0.001; **** = *p* < 0.0001

### 2.5. Ethics Statement

The experiments, maintenance, and care of fish complied with the guidelines of the European Convention for the Protection of Vertebrate Animals used for Experimental and other Scientific Purposes (CETS No. 123). The animal studies were approved by the local ethics committee of the University of Liège, Belgium (Laboratory accreditation No. 1610008). All efforts were made to minimize suffering of the fish.

## 3. Results

### 3.1. Confirmation of CyHV-2 Presence in Pooled Organ Samples

**Figure 1:**
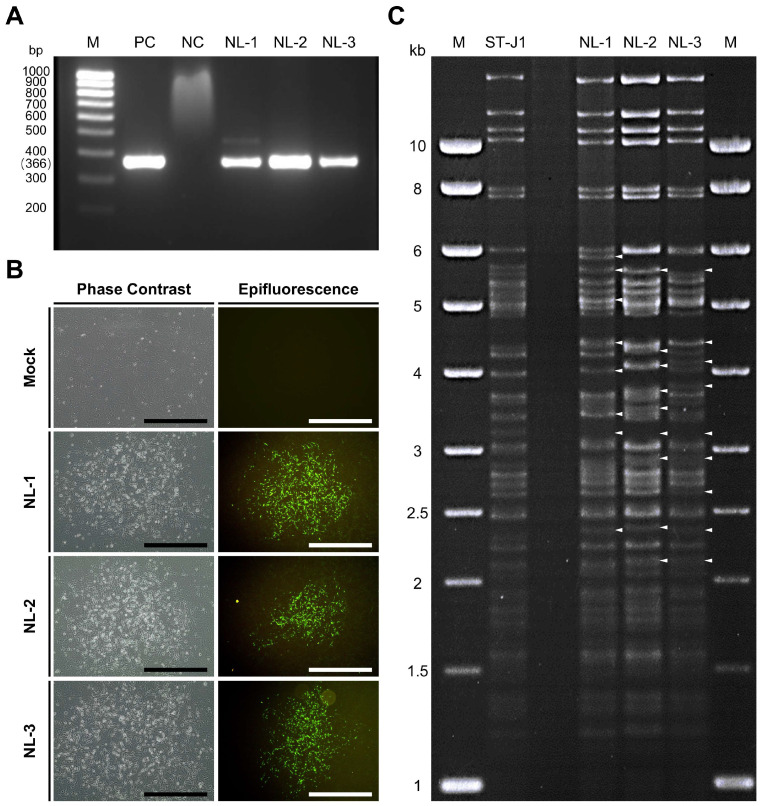
(**A**) PCR screening for CyHV-2 in three samples from fish from The Netherlands. M: molecular weight marker (bp: base pair); PC: positive control (from ST-J1); NC: negative control; NL-1: NL-1 tissue DNA; NL-2: NL-2 tissue DNA; NL-3: NL-3 tissue DNA. (**B**) CPE imaging via microscopy at 10 dpi. The left and right panels represent the brightfield and epifluorescence channels, respectively, with different representative plaques shown in each channel. CPE was only observed in RyuF-2 cells inoculated with purified tissue supernatant from NL-1 #0, NL-2 #0, and NL-3 #0, and was entirely absent from the mock control. Black scale bar = 600 µm; white scale bar = 500 µm. (**C**) Genomic analysis of CyHV-2 strains. Viral DNA from plaque-purified sub-cultures from ST-J1, NL-1, NL-2, and NL-3 (one from each) were compared by RFLP analysis after digestion using the SacI restriction enzyme. The white arrowhead indicates some of the most notable differences relative to the ST-J1 reference strain. M: molecular weight maker (kb: kilobase).

Three pooled organ samples from diseased fish initially suspected of being infected with CyHV-2 were generously provided by WBVR (Wageningen Bioveterinary Research), part of Wageningen University in The Netherlands. We assigned them the names NL-1, NL-2, and NL-3 (see Section 2 for details). Upon arrival in our lab, these samples were screened for the presence of CyHV-2 using a PCR assay targeting the CyHV-2 helicase gene. Through this process, we confirmed the presence of the virus, with the expected 366 bp PCR product forming in all three samples (Figure 1A).

In parallel, RyuF-2 fresh cells were inoculated with dilutions of purified supernatant prepared from the NL-1, NL-2, and NL-3 pooled tissue samples (passage #0 or p#0; see Section 2) and incubated at 25 °C. At 10 dpi, the characteristic morphology of CyHV-2 associated CPE became visible via brightfield phase contrast microscopy and eventually formed plaques. The observed characteristics included focal areas of granulation, cell vacuolization, and the emergence of rounded phase-bright cells (Figure 1B left panel). By approximately 14 dpi, infected cells in these plaques began to detach, with this process starting from the central regions and radiating outwards as time progressed. The same CPE-like and plaque morphology was not observed in the mock group. Notably, no syncytial plaque formation was observed in cells infected with any of the three isolates.

These regions exhibiting signs of CyHV-2-induced CPE and plaque formation via brightfield phase contrast microscopy also stained positive for CyHV-2 via indirect immunofluorescent staining and epifluorescence microscopy (Figure 1B, right panel). This indicated (i) that the p#0 supernatant samples prepared from the CyHV-2 PCR-positive samples contained infectious CyHV-2 virus particles and (ii) that these three CyHV-2 isolates, NL-1, NL-2, and NL-3 could be cultured in vitro.

### 3.2. Virus Isolation and Amplification

While the initial in vitro culture of the p#0 supernatant led to the formation of CPE and plaques, for any of these new isolates to be useful as part of future CyHV-2 in vivo infection models, it was crucial that they could be stably passaged in cell culture. Subsequent in vitro passaging of these three isolates revealed significant differences in the in vitro propagation of NL-1, NL-2, and NL-3 in RyuF-2 cells. Only NL-1 and NL-2 isolates could be consistently passaged successfully, reaching up to ten passages (#10). Conversely, the NL-3 isolate could not be passaged beyond p#3 in RyuF-2 cells, with cytopathic effects disappearing in the subsequent passages.

### 3.3. Genomic and Phylogenetic Analysis

At p#1, three independent plaques were sub-cultured from each isolate, amplified for one passage in cell culture flasks, generating p#2. At 10 dpi, viral culture media was used to prepare viral supernatant for subsequent virus purification and for extraction of pure viral DNA (i.e., free of host cell DNA), which would be suitable for genomic analysis. In addition, the CyHV-2 reference strain ST-J1 was used as a reference for comparative analysis.

Since the samples originated from distinct geographic regions and hosts, we hypothesized that the NL-1, NL-2, and NL-3 CyHV-2 isolates may represent genetically distinct CyHV-2 lineages. To investigate this further, we used RFLP to conduct a quick preliminary analysis on one plaque-purified sub-culture from each isolate. The results are presented in Figure 1C. This indicated that the NL-1, NL-2, and NL-3 exhibited major genetic differences relative to the ST-J1 strain. While some of these differences were common to two or more of these isolates, indicating that they were more closely related to each other than to the ST-J1 reference, the results also indicated notable differences between the three isolates, indicating that they were also somewhat genetically distinct from each other.

**Figure 2:**
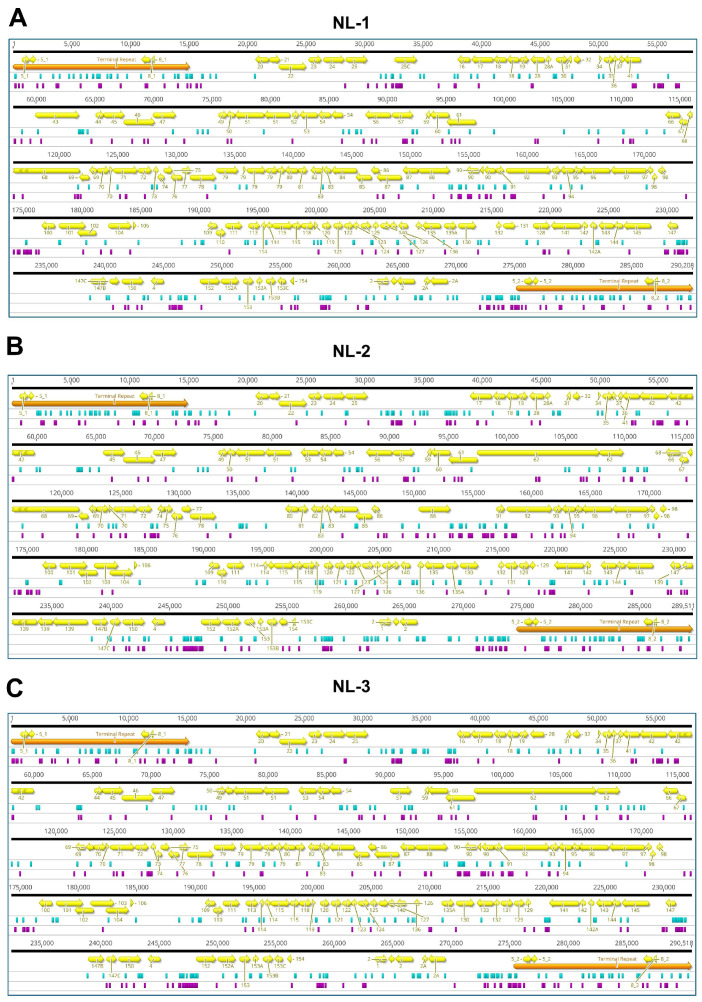
Schematic of (**A**) NL-1, (**B**) NL-2, and (**C**) NL-3 assembled genomes. Direct terminal repeats are indicated in orange. ORFs with 99% sequence match to ORFs in CyHV-2 ST-J1 reference genome (NC_019495.1) are indicated in yellow and labelled by their corresponding ORF number in the reference genome. Substitutions involving more than one nucleotide, insertions, or deletions are highlighted in turquoise. SNVs are indicated in purple. The locations of all variants are based on their starting point in each of their respective genomes, with feature lengths enlarged to ensure visualization on the map (coordinates correspond to the data in Tables S1–S3).

**Figure 3:**
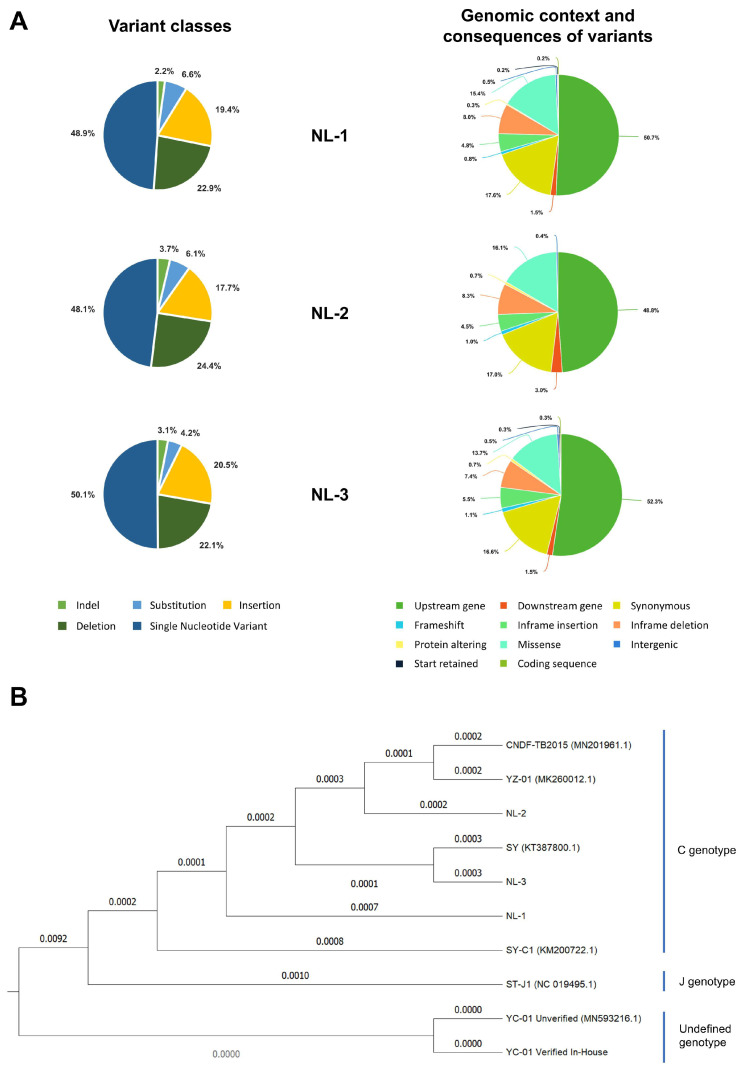
Genomic comparison of isolates to other CyHV-2 strains (**A**) (Left) Pie charts indicating the breakdown of different variant classes that were identified in each of the newly sequenced isolates relative to the CyHV-2 ST-J1 reference strain. Variant classes are defined as per https://www.ensembl.org/info/genome/variation/prediction/classification.html (accessed on 1 April 2025). (Right) Pie charts indicating the breakdown of corresponding genomic context and consequences of all variants identified. Consequence types are as defined by the Sequence Ontology (SO) project and are summarized here: https://www.ensembl.org/info/genome/variation/prediction/predicted_data.html (accessed on 1 April 2025). (**B**) Phylogenetic tree based on complete genome sequences of CyHV-2 strains. Values above or below the branches represent the number of substitutions per site. The tree is rooted using the undefined genotype clade as an outgroup.

To determine the exact nature of the differences, viral DNA prepared from purified particles of each NL-1, NL-2, and NL-3 were also subject to genomic sequencing. As part of this process, three independent plaque-purified sub-cultures (or replicates) were sequenced from each isolate. Genome assembly revealed little or no differences between the plaque-purified sub-cultures of individual isolates. For each isolate, the sequences of the three purified sub-cultures were used to generate a single representative consensus genome sequence, which was used in further analysis. The representative genome sequences for the NL-1, NL-2, and NL-3 isolates are available on GenBank under the accession numbers PQ738159.1, PQ723683.1, and PQ738160.1, respectively. The results of genome sequencing were in agreement with the RFLP analysis, indicating that each isolate exhibited many differences (each exhibiting > 600 differences) relative to the ST-J1 reference strain. These are presented in Figure 2, described in detail in Tables S1–S6, and summarized in Table 1. Some of these differences to the reference strain included major changes, such as frameshifts in several ORFs of unknown function; however, as expected, most of the changes consisted of single nucleotide variants (SNVs) (Figure 3A left and Table 1) and either occurred outside of protein coding regions or were synonymous mutations, therefore having no impact on the amino acid sequence (Figure 3A Right).

The high number of differences to the ST-J1 reference (Table 1, Figure 2 and Figure 3A) and the commonalities among the isolates observed in the RFLP analysis (Figure 1C) indicated that these newly sequenced isolates were more related to each other than to the ST-J1 strain. To examine this further, a phylogenetic tree was constructed based on sequences of all currently available CyHV-2 genomes and these newly sequenced isolates (Figure 3B). This analysis indicated that, indeed, the new NL-1, NL-2, and NL-3 isolates were more closely related to each other than to the ST-J1 reference strain, the latter of which represents the J genotype [42]. Notably, the three new isolates are more related to the C genotype strains [42,43]. Within this large C genotype clade, NL-1, NL-2, and NL-3 occupy separate sub-clades, indicating that they are genetically distinct from each other. As a positive control for our genome assembly workflow, we also assembled the YC-01 genome. Our YC-01 assembly was almost identical to the publicly available YC-01 genome sequence, with notable genomic rearrangements and inversions relative to other strains [44], which is currently described as “Unverified” in GenBank. Indeed, the YC-01 strain is more distantly related to all other fully sequenced strains (~96.7% vs. >97% identity to the CyHV-2 reference sequence) and may represent a new distinct CyHV-2 genotype clade, which we refer to as “Undefined” (Figure 3B).

### 3.4. Pathogenicity of the Three Isolates

**Figure 4:**
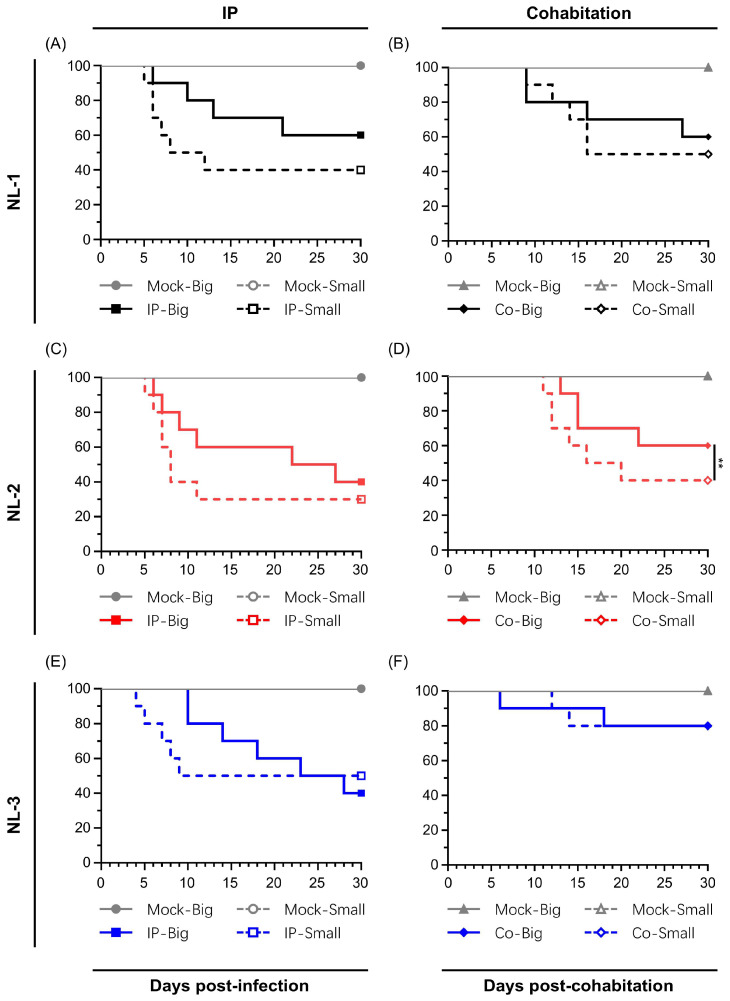
Primary comparison of inherent in vivo pathogenicity of the three isolates (**A**,**B**) NL-1, (**C**,**D**) NL-2 and (**E**,**F**) NL-3. The pathogenicity of the indicated strains was tested using adult Shubunkin goldfish (big, average weight 12 ± 1.4 g) and the young adult Shubunkin goldfish (small, average weight 5.84 ± 0.6 g). Each group consists of 10 subjects. Fish were either mock-infected or infected via IP injection with the respective strains (100 µL in big fish, 50 µL in small fish). For the cohabitation trial, at 5 days post-injection, 10 more fish were added to each tank. The fish were monitored daily for clinical signs of CyHV-2 disease, and fish reaching the endpoints were euthanized. In all graphs, the y-axis represents percentage survival.

In order for any of the newly sequenced isolates to be useful in terms of utilization as a robust CyHV-2 in vivo infection model, it was important to first determine if the p#0 inoculums were actually capable of causing clinical disease and mass mortality in commercially and economically relevant goldfish breeds. As part of this preliminary assessment of the virulence of these new isolates, we conducted in vivo challenge experiments using an in-house batch of adult Shubunkin goldfish. In this trial, two sizes of adult Shubunkin goldfish were used. These were referred to as “small” and “big”. Fish in the “big” group were twice the mean weight of those in the small group and thus received twice the volume of the non-titered p#0 inoculum relative to the small group. Subjects were inoculated either by intraperitoneal (IP) injection or by cohabitation with IP injected fish (Figure 4). This initial exploratory experiment was limited to 10 fish per group due to the low amounts of p#0 infectious material that was available.

Mortality rates among the NL-1 and NL-2 groups were similar, with both the IP and cohabitation groups showing high mortality, particularly in small fish. The IP-injected fish exhibited a more rapid onset of illness and clinical symptoms, with mortality in small fish primarily occurring between 5 and 12 dpi (60% for NL-1, 70% for NL-2). Big fish had a longer disease course, higher survival rates and delayed onset of death, relative to that of small fish (Figure 4A,C). Sentinel fish cohabitating with IP-injected fish exhibited lower mortality rates of 50% for NL-1 and 60% for NL-2 in small fish, both higher than the 40% seen in big fish, with NL-1 deaths concentrated between 9 and 16 dpi and NL-2 between 11 and 20 dpi (Figure 4B,D). Despite the consistent differences in mortality between big and small fish inoculated with NL-1 and NL-2, due to the limited sample size in this preliminary experiment, these differences were only statistically significant for groups inoculated with NL-2 by cohabitation (Figure 4D).

The patterns observed in the NL-3 group were different than the NL-1 and NL-2 groups. In big fish, the NL-3 inoculum induced slightly more mortality than the NL-1 and NL-2 inoculums. However, it induced similar mortality in small fish (Figure 4E). Furthermore, in contrast to NL-1 and NL-2, in cohabitation experiments with NL-3, only a limited mortality of 20% was observed among both small and big sentinel fish (Figure 4F). No statistically significant difference was observed between big and small fish inoculated with NL-3 via the routes tested. Moreover, even though one early mortality was recorded among the big sentinel fish, this mortality was not found to be attributed to CyHV-2 infection.

However, regardless of the isolate that was used, in all IP infected groups, the fish exhibited classical clinical symptoms, including diminished appetite, lethargic swimming velocity, protruding eyeballs, distended abdomen, drooping of dorsal fin, and loss of balance, with only very few minor instances of hemorrhaging on the skin. Post-mortem examinations on subjects undergoing mortality during the experiment revealed severe ascites, enlarged spleen and kidneys, and white dot-like nodules within the kidneys, while the intestines were swollen with no food material remaining, and no abnormalities were found in the gills (Figure S1). Sentinel fish from the cohabitation experiment exhibited similar but less severe clinical symptoms. No clinical symptoms, deaths, or CyHV-2 PCR positive results were observed in the mock group.

**Figure 5:**
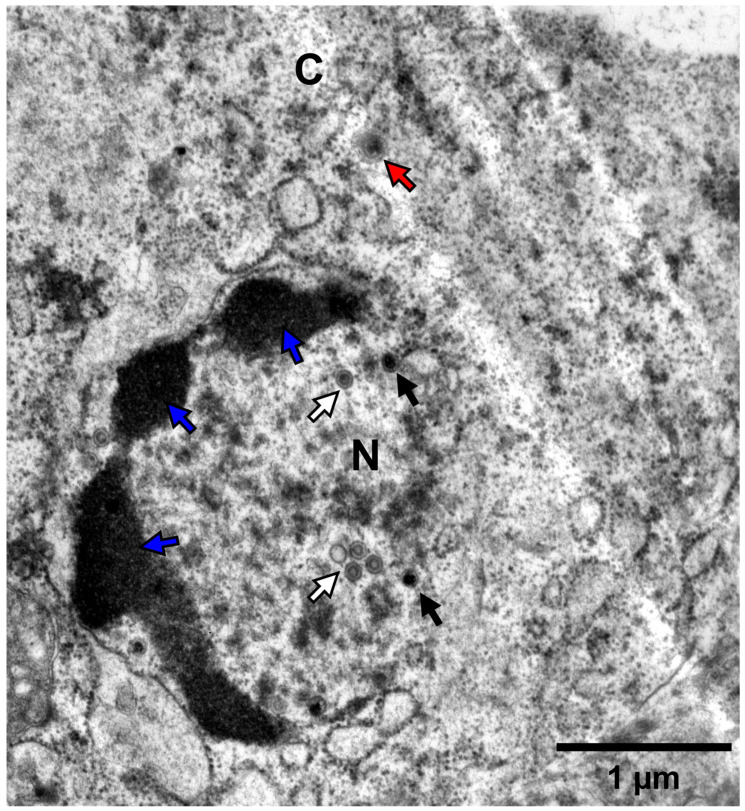
Transmission electron microscopy of the CyHV-2 NL-2 isolate. This image represents the ultrastructure of CyHV-2-infected kidney cells. Nuclear chromatin margination consistent with herpesvirus infection is visible (blue arrows). Virus particles at different stages of assembly are also visible, including some prime examples of incomplete nuclear particles with empty or partially complete cores (white arrows) and capsids with a central electron-dense core (black arrows). Cytoplasmic virions, which appear to be larger than nuclear viral particles and thus potentially organelle-enveloped as part of the process of egress from the cell, are also visible (red arrows). N = nucleus; C = cytoplasm.

Kidneys from fish that underwent mortality were promptly sampled and stored at -80 °C, whereas kidneys from surviving fish were collected at the end of the experiment. DNA was extracted from all kidneys, and subsequent PCR testing confirmed the presence of CyHV-2 in all infected subjects (data not shown). Separately, some kidney samples from fish infected with the NL-2 isolate (IP injection) were immediately fixed after sampling for later analysis using high-magnification transmission electron microscopy. This provided clear evidence of herpesvirus replication and assembly in kidney tissue (Figure 5). Infected kidney cells exhibited nuclear changes characterized by central nucleoplasmic transparency and marginal chromatin aggregation. The nucleus contained aggregates of characteristic naked herpes-like virus nucleocapsids (90–100 nm), including incomplete particles with empty or partially complete cores and capsids with a central electron-dense core. Potential organelle-enveloped virus particles were also observed. These ultrastructural characteristics of the virus particles are consistent with those of CyHV-2 virions described in other reports [45,46,47]. Furthermore, the virus was successfully re-isolated from the kidneys in cohabitating goldfish (data not shown).

### 3.5. In Vitro Growth Kinetics Comparison

**Figure 6:**
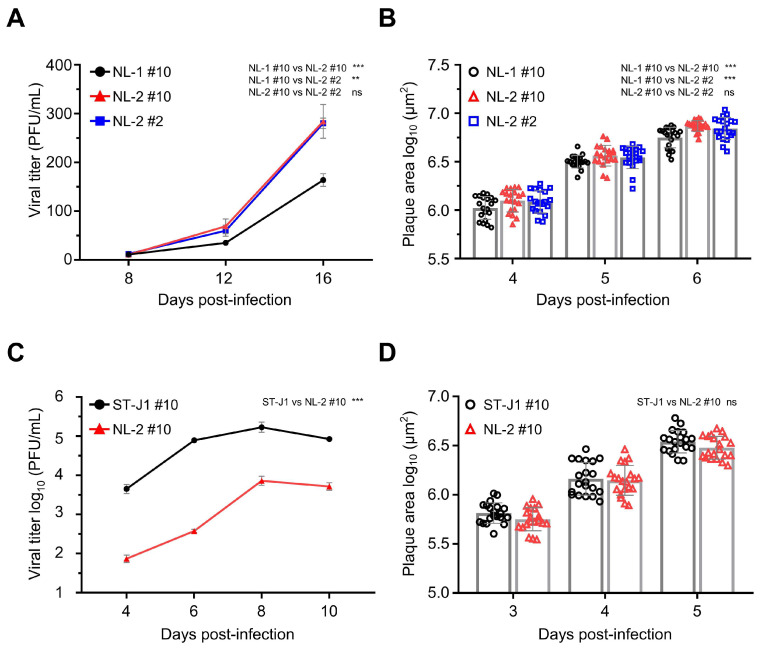
Comparison of in vitro growth kinetics and plaque sizes between the NL-1 and NL-2 isolates. (**A**) Viral growth curve comparison between NL-1 #10, NL-2 #10, and NL-2 #2. RyuF-2 cells were infected with the indicated strains (MOI = 0.0001), and the log_10_ value of the titer (PFU/mL) in the supernatant was determined at 8, 12, and 16 dpi. Data represent the mean ± SEM of triplicate measurements. (**B**) Viral plaque size comparison between NL-1 #10, NL-2 #10, and NL-2 #2. RyuF-2 cells were infected with the respective strains, and plaque areas were measured at 4, 5, and 6 dpi. Data represent the mean ± SEM of 20 individual plaques. (**C**) Viral growth curve comparison between ST-J1 and NL-2. RyuF-2 cells were infected with the indicated strains (MOI = 0.01), and the log_10_ value of the titer (PFU/mL) in the supernatant was determined at 4, 6, 8, and 10 dpi. Data represent the mean ± SEM of triplicate measurements. (**D**) Viral plaque size comparison in ST-J1 and NL-2. RyuF-2 cells were infected with the respective strains, and plaque areas were measured at 3, 4, and 5 dpi. Data represent the mean ± SEM of 20 individual plaques.

All of the p#0 samples were shown to contain infectious CyHV-2 capable of causing clinical disease in the Shubunkin goldfish model. However, to be useful in future studies, it was important that these isolates could also be easily cultured in vitro in order to be easily titrated and to facilitate the sufficient expansion of viral stock for in vivo studies. In general, the ability of a viral isolate to replicate in vivo is not indicative of its ability to replicate in vitro or adapt quickly to this specific biological niche, and there are many factors that influence this. For example, while causing clinical disease in vivo, as described earlier, the passaging of the NL-3 isolate could not be sustained beyond p#3 in vitro, indicating that NL-3 was not practical for future use as a robust CyHV-2 in vivo infection model. Therefore, we proceeded with further characterization of NL-1 and NL-2 only, which, in contrast to NL-3, grew well in vitro, with viral titers increasing over several passages, thus providing sufficient viral stock for further characterization. Notably, the NL-2 reached higher titers faster, indicating that it might be better adapted to in vitro culture. To investigate this further, we compared the in vitro growth kinetics of NL-1 and NL-2. Importantly, to maximize the validity of the comparison, in addition to using the same dose of each isolate (MOI = 0.0001) for viral growth curves, we also compared them at the same passage number, in this case p#10, (referred to as NL-1 #10 and NL-2 #10, respectively). The results indicated that viral titers remained low initially, from 8 to 12 dpi. However, from 12 dpi to 16 dpi, the titers of both isolates increased rapidly. At the end of the experiment, at 16 dpi, NL-2 CyHV-2 reached titers close to 300 PFU/mL, nearly twice that of NL-1 #10, and we observed a significant difference in the growth kinetics of these two isolates at p#10, indicating that, indeed, NL-2 is better suited to in vitro culture compared to NL-1 (Figure 6A).

To explore the possibility that the superior growth kinetics of NL-2 may have been simply due to incremental adaptation to in vitro culture during the previous 10 passages, in parallel, we included a growth curve for NL-2 at passage #2. This indicated that there was no significant change growth kinetics of NL-2 between early (p#2) and later (p#10) passages (Figure 6A). Similar patterns were also observed when comparing plaque size between the same isolates, with plaques from NL-2 #10 being slightly but significantly larger than NL-1 #10, with no difference between NL-2 #2 and NL-2 #10 in terms of plaque size. (Figure 6B). Taken together, these experiments indicated that out of the three new isolates, the biological properties of NL-2 made it intrinsically better suited to stable and efficient culture in vitro and therefore potentially represented a more useful candidate for utilization in a more robust CyHV-2 in vivo infection model.

Next, we compared the in vitro growth kinetics NL-2 #10 with the CyHV-2 reference strain ST-J1, also at p#10, using an MOI of 0.01 for both. At this MOI, the progression of the viral growth curve over time was similar for both strains, with viral titers increasing over the first three timepoints, peaking at 8 dpi, and decreasing by 10 dpi (Figure 6C). However, the viral titer of ST-J1 was higher than NL-2 at each timepoint, resulting in a significant difference between the two strains overall, demonstrating that relative to NL-2, the ST-J1 strain exhibits superior replication kinetics in vitro. Despite this, the comparison of viral plaque size at early timepoints in the infection (3, 4, and 5 dpi) revealed no consistent difference between the two strains, with no statistically significant difference overall (Figure 6D).

Taken together, these experiments indicated that NL-2 was better suited to in vitro culture compared to NL-1 and NL-3, and furthermore, its properties remained stable over multiple passages. While the ST-J1 reference strain exhibited superior in vitro replication properties, the replication kinetics of the NL-2 isolate was sufficient to build up enough viral stock to facilitate its use in subsequent in vivo experiments.

### 3.6. Comparison of In Vivo Pathogenicity of CyHV-2 NL-2 Strain and Reference Strain ST-J1

**Figure 7:**
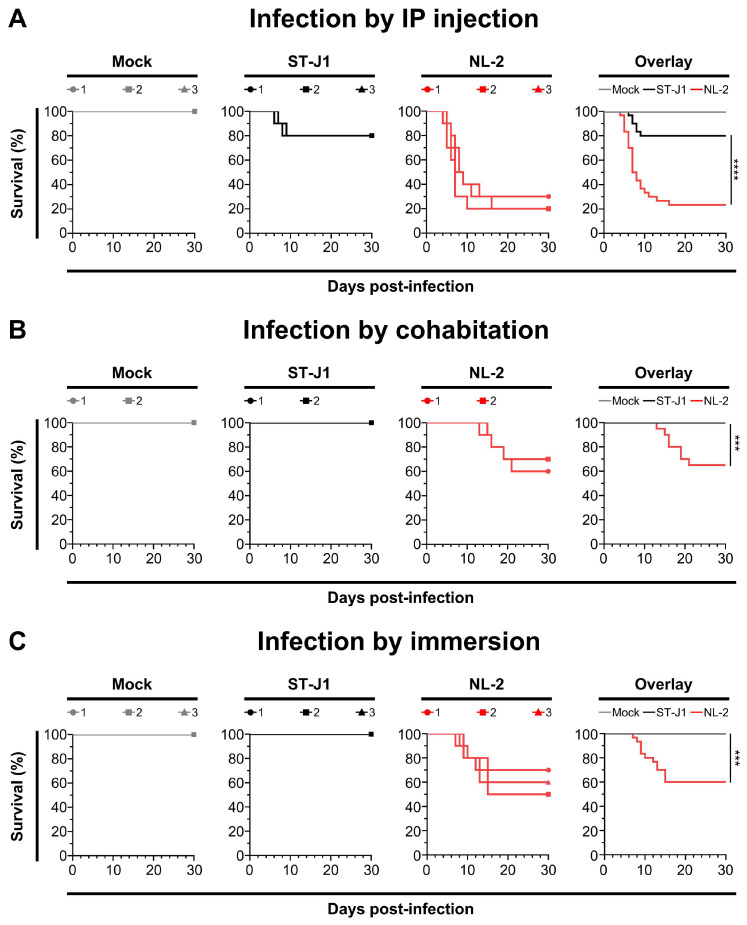
Comparison of NL-2 and ST-J1 in young adult Shubunkin goldfish in vivo. The pathogenicity of the two strains (both at passage #10) was tested in young adult Shubunkin goldfish (average weight 5.84 ± 0.6 g). (**A**) Comparison of in vivo pathogenicity of the ST-J1 and NL-2 strains after infection by IP injection. Fish were mock-infected or infected by IP injection (triplicate groups each consisting of 10 subjects) with the indicated strains. Each subject received a 50 µL injection of inoculum (1000 PFU/fish). The fish were monitored daily for clinical signs of CyHV-2 disease, and fish reaching the endpoints were euthanized. In the overlay graph, the mean survival results were compared. (**B**) Comparison of in vivo pathogenicity of the ST-J1 and NL-2 strains after infection by cohabitation. The same number of goldfish were placed as sentinel fish in the first two replicates of each group in the experiment described in (**A**) (5 days after infection by IP injection). Fish were examined daily, and those reaching the endpoints were euthanized. In the overlay graph, the mean survival results were compared. (**C**) Comparison of in vivo pathogenicity of the ST-J1 and NL-2 strains following infection by immersion. Fish were mock-infected or infected by immersion in water containing each virus strain at a final concentration of 2000 PFU/mL (triplicate groups each consisting of 10 subjects). Fish were examined daily, and those reaching the endpoints were euthanized. In the overlay graph, the mean survival results were compared.

After examining the in vitro growth kinetics, we compared the virulence of the NL-2 strain to that of the CyHV-2 reference strain ST-J1 in vivo. In this experiment, young adult fish were inoculated with the same dose of each CyHV-2 strain via IP injection, allowing a valid comparison between both strains. Analysis of the survival curves indicated a significant difference between the strains in terms of mortality rates (Figure 7A). NL-2 induced more intense and faster-developing clinical symptoms associated with CyHV-2 disease compared to ST-J1. Symptoms in the NL-2 group began at 4 dpi, with mortality starting at 5 dpi, rapidly increasing after, and finally ceasing approximately two weeks post-infection, with an average mortality rate of 76.6%. In contrast, the ST-J1 group exhibited only mild symptoms and a short recovery period, with mortality events limited to between 6 and 9 dpi and a significantly lower average mortality rate of 20%. No mortality was observed in the mock infected groups.

The cohabitation experiments also revealed a big difference between the two CyHV-2 strains, with only sentinel fish in the NL-2 group exhibiting mortality after cohabitation with IP-injected fish. Mortality among sentinel fish occurred mainly between 2 and 3 weeks post-cohabitation (Figure 7B), leading to an overall mortality rate of approximately 35%, which was lower than that of the IP-inoculated group. Notably, this pattern, including the mortality window, was consistent with the earlier NL-2 cohabitation experiment (Figure 4D), but the mortality rates were slightly lower in this later cohabitation experiment. In stark contrast to this, no mortality or clinical disease was observed in the ST-J1 cohabitation group or the mock group (Figure 7B).

We observed a similar contrast between the two strains in an additional experiment, which involved inoculation by immersion in water containing virus. This involved the immersion of 10 subjects in 1L of water containing 2000 PFU/mL of each strain, for a period of 2 h, before being returned to 60 L tanks. Unlike IP injection, this mode of inoculation is more representative of the natural waterborne infection route. Using this inoculation strategy, the NL-2-inoculated fish exhibited an average mortality rate of approximately 40%, which occurred during one to two weeks post-infection (Figure 7C). As per earlier experiments, the typical clinical symptoms such as abdominal swelling were observed, but more severe skin lesions were observed in fish from the NL-2 infection group. Moribund fish exhibited multiple skin hemorrhages and enlarged kidneys (Figure S2A). In contrast to the earlier experiments, a white necrosis appeared on the tail fins of all goldfish three days after infection, with some fish experiencing tail fin structure destruction and impaired swimming posture in the later stages of infection (Figure S2B). In contrast, no mortality or clinical disease was observed within the ST-J1 group of fish. Taken together, these experiments indicated that the NL-2 isolate was much more suitable for in vivo infection experiments in European-sourced Shubunkin goldfish populations, compared to the ST-J1 CyHV-2 reference.

## 4. Discussion

A crucial element in the study of any virus is the identification of a suitable virus–host model for in vivo experimental infection. Being able to study a virus in its natural host facilitates more biologically relevant insights into many aspects of virus–host interaction. Like all known members of the genus *Cyvirus*, CyHV-2 can be easily studied in its natural hosts, including goldfish, making them interesting models to use in the study of herpesvirus infections, with strong relevance to other vertebrate species. Indeed, this process is greatly facilitated by the availability of a host that can be easily bred and maintained under controlled conditions to allow a consistent and sufficient supply of subjects for experiments.

With regard to CyHV-2, there are many breeds of goldfish that meet these criteria, including Shubunkin goldfish. In addition to being susceptible to CyHV-2, Shubunkin goldfish are heavily associated with international trade [16], which is likely to be a key epidemiological factor in the global spread of CyHV-2, making them relevant host models in the context of research into disease control and mitigation. However, while the CyHV-2 ST-J1 strain can induce mortality in European-sourced adult Shubunkin goldfish populations by IP injection, these populations exhibit no mortality when challenged with this virus strain by immersion (Figure 7), with similar observations after YC-01 CyHV-2 challenge [15]. However, it also remains possible that ORF disruptions in these CyHV-2 strains—which may or may not have emerged during passaging [7,44]—may also be responsible for this lack of virulence, which we will discuss later. Regardless of whether this is due to host or viral factors, this renders these CyHV-2 strains sub-optimal to use, and with these, a wide range of economically and epidemiologically relevant cohorts. At the same time, outbreaks of CyHV-2 among European populations of Shubunkin goldfish indicate that they remain susceptible to new and uncharacterized CyHV-2 variants [16]. We therefore sought to identify and characterize some of these new CyHV-2 isolates, in order to assess their utility in future CyHV-2 studies utilizing the Shubunkin goldfish infection model.

In Europe, where CyHV-2 has a wide geographical distribution, CyHV-2 outbreaks are frequently described in goldfish and gibel carp populations in both aquaculture facilities and in the wild [45,48,49,50,51,52]. Like other regions within Europe, The Netherlands has experienced outbreaks as early as 2011, with repeated occurrences since then [53]. As per patterns across the globe, these represent a mixture of cases in both domesticated and wild fish [16,17,54]. Given the frequency of CyHV-2 detection in The Netherlands, it represented an ideal source from which to obtain high quality tissue samples from diseased fish for further investigation, ultimately leading to the isolation of three new isolates, NL-1, NL-2, and NL-3. Further in vitro and in vivo characterization established that the NL-2 isolate (*i*) could be stably cultured in cells, leading to the production of a viral stock, and (*ii*) exhibits much greater virulence in adult Shubunkin goldfish relative to the CyHV-2 ST-J1 reference strain. Given their properties, the use of this CyHV-2 NL-2 isolate in combination with Shubunkin goldfish represents a more optimal in vivo infection model compared to the use of the CyHV-2 ST-J1 reference strain. Thus, this newly characterized in vivo infection model represents an ideal tool to use in future research into CyHV-2 disease mitigation, control, and viral host interaction.

Whole genome sequencing of the NL-1, NL-2, and NL-3 isolates and genetic comparison of these strains to existing strains indicated that they are distantly related to the CyHV-2 ST-J1 reference strain and the CyHV-2 YC-01 strain, both of which cause limited or no mortality in adult Shubunkin goldfish when exposed to virus by cohabitation and/or immersion (see [15] and Figure 7). Instead, these new isolates are more related to other CyHV-2 strains that occupy the C genotype clade (Figure 3B). Furthermore, these three new isolates are also genetically distinct from each other and thus occupy separate sub-clades within the C genotype clade. However, the genetic differences between these new isolates and the ST-J1 reference strain may potentially provide some interesting insights into phenotypic differences observed in further characterization (discussed in more detail later).

The initial in vivo characterization of these new CyHV-2 isolates revealed that they could all cause clinical disease associated with CyHV-2 in adult Shubunkin goldfish subjected to IP inoculation. In all cases, this also led to the subsequent transmission of virus to cohabiting fish (Figure 4). While this experiment was conducted using unknown titers of each isolate, making it difficult to make a valid comparison of virulence between the strains, importantly, it did indicate that they were capable of inducing mortality in adult Shubunkin goldfish. Notably, in this experiment, the results with NL-1 and NL-2 were consistent with our previous observations with the CyHV-2 Shubunkin goldfish model [15], which indicated that earlier developmental stages are more permissive of CyHV-2 replication and exhibit higher mortality rates. However, again, NL-3 was slightly different in this regard; while mortality certainly occurred earlier and faster in smaller fish in this group, it ceased very early. Taken together these experiments demonstrated that the p#0 inoculums prepared from NL-1, NL-2, and NL-3 tissue homogenates contained infectious CyHV-2, capable of causing clinical disease and mass mortality in the Shubunkin goldfish model, and therefore, we concluded that they may represent ideal candidates to replace CyHV-2 ST-J1 or YC-01 strains in this in vivo infection model, as the latter cause relatively little disease after infection by immersion.

However, in addition to causing virulence in vivo, for these new viral isolates to be useful, it was equally important that they could be stably and efficiently passaged in vitro. The initial in vitro culture of the three CyHV-2 isolates revealed slow CPE development in RyuF-2 cells post-inoculation (0 to #1), with virus plaques identified after approximately 10 days (Figure 1B). This was similar across the three isolates and consistent with the previous isolation of other CyHV-2 strains [45,55,56,57]. However, differences between the isolates emerged during continuous passage. Most notably, we observed that CPE caused by NL-3 could not be observed beyond the third passage. While this does not mean that the virus is totally absent after the third passage, it does indicate that the initial NL-3 phenotype may be intrinsically unstable in vitro. It also suggests that high levels of productive lytic infection may be limited to early passages only when using the NL-3 isolate. Similar instances of instability of CyHV-2 isolates in vitro have been observed using KF-1 cell lines [45,57] and the goldfish-derived cell lines GFSe and GFKf [58]. This suggests that the NL-3 isolate is intrinsically less fit in vitro compared to the other isolates. Alternatively, while it could not be passaged continuously in RyuF-2 cells, it remains possible that it exhibits greater fitness in other cells that are permissive to CyHV-2 replication. For example, in previous studies, it was observed that some CyHV-2 isolates were capable of causing CPE in EPC cells, while others were not [10]. However, it is also important to consider experimental differences between these studies that may also contribute to these differences. In contrast to NL-3, the NL-1 and NL-2 isolates could be passaged more stably in RyuF-2 cells, with NL-2 exhibiting superior replication kinetics relative to NL-1 in this cell line. However, the CyHV-2 ST-J1 reference strain exhibited much higher replication kinetics, compared to NL-2. Taken together, these observations allow us to infer a simple ranking system in terms of replication kinetics or fitness in RyuF-2 cells, i.e., “ST-J1 > NL-2 > NL-1 > NL-3”.

The genetic determinants of these different phenotypes are unclear as each isolate exhibits hundreds of mutations relative to the ST-J1 reference strain (Figure 2, Tables S1–S3). Most of these are SNVs that occur either outside of ORFs or result in synonymous changes within ORFs, with no impact on protein sequences (Figure 3). However, other mutations, such as insertions and deletions, can cause frameshifts within ORFs, and hence, these may be much more likely to result in phenotypic impacts (Tables S4–S6). Notably, all of the new isolates have frameshifts in ORF138, ORF151A, ORF25B, and ORF156, relative to ST-J1. The frameshifts in ORF138 and ORF25B are identical in all of the new isolates and are also present in other CyHV-2 strains. The frameshifts in ORF156 are identical in NL-1 and NL-3, and a very similar frameshift is present in NL-2 ORF156, leading to a similar ORF156 truncation. These common mutations point towards a monophyletic origin of these three new isolates separate from the J genotype. Conversely, the ORF151A frameshifts in the NL-1, NL-2, and NL-3 isolates are not identical to each other, and only the NL-2 version of ORF151A is found in other CyHV-2 strains. It is possible that the ST-J1 proteome is more optimum for CyHV-2 fitness in vitro. Outside of these four major differences, which are common to all three isolates, additional changes relative to this “optimum proteome” may further reduce fitness in vitro. Consistent with this, it is notable that NL-3, which exhibited the least fitness in vitro, has more frameshifts (a total of eight, including frameshifts in ORF28A, ORF30, ORF89, and ORF90) relative to ST-J1 than the other two isolates. Thus, the NL-3 proteome, which is predicted to be more divergent from that of ST-J1 than those from the other two isolates, may be less optimal for CyHV-2 fitness in vitro. However, NL-2 proteome, exhibiting seven frameshifts relative to ST-J1, is also quite divergent from the ST-J1 proteome, yet can be stably passaged in vitro. This raises the possibility that the specific ORFs disrupted in NL-3 may be important for facilitating stable passaging of CyHV-2 in vitro, and it may be interesting to investigate this further in future studies. However, it is important to note that outside of highly disruptive changes such as frameshifts, it remains possible that the smaller protein changes, such as single nucleotide changes leading to non-synonymous amino acid substitutions, may also impact fitness in vitro, as we described recently in the closely related virus, *Cyvirus cyprinidallo3* [59] (or CyHV-3 formerly known as *Cyprinid herpesvirus 3* [4]).

The direct comparisons between the CyHV-2 NL-2 isolate and the CyHV-2 reference strain ST-J1 in vitro and in vivo, also yielded some interesting results. Despite the ST-J1 being more fit in vitro, it was less fit in vivo, and vice versa for NL-2. This is similar to our previous observations with different CyHV-3 strains [59,60]. This may occur as a consequence of continuous passaging in vitro, which introduces a selective pressure that favors variants that are adapted to in vitro conditions. In the absence of any selective pressure to retain in vivo fitness, these in vitro adapted strains may develop an attenuated phenotype in vivo, as we described previously with CyHV-3 variants [59]. It is unclear if the same has occurred with ST-J1, leading to an attenuated phenotype in some goldfish breeds (or populations thereof). Notably, there were no genomic differences between the original ST-J1 stocks used to generate a viral stock for these experiments and the ST-J1 reference sequence in GenBank (data not shown).

Interestingly, a total of seven frameshifted ORFs exist in NL-2 relative to the ST-J1 strain. These include ORF1, ORF16, ORF25B, ORF52, ORF138, ORF151A, and ORF156 (Table S5). In the case of NL-2 ORF1, while a frameshift variant is present at the start of the coding sequence, as the start codon is still retained, there is actually no impact on the protein sequence. Given that these represent the biggest differences in their respective proteomes, it may be interesting to determine if ST-J1 virulence in vivo may be improved through replacement of one or more of these with the equivalent NL-2 allele(s). With the exception of ORF156, the functions of the proteins encoded by these CyHV-2 ORFs have been predicted based on homology with other proteins in related viruses such as CyHV-3 [7], which may allow us to hypothesize which of these changes are more likely to be responsible for these differences in phenotype between ST-J1 and NL-2 in vivo.

Some of these frameshifts result in the truncation of predicted proteins in NL-2 relative to ST-J1 and other CyHV-2 strains, some of which may be responsible for the reduced fitness of NL-2 relative to ST-J1 in vitro. For example, ORF52 is predicted to encode a signal peptide that is truncated in NL-2 relative to ST-J1 and most other fully sequenced strains. However, the potential functional impact of this truncation on NL-2 fitness in vitro relative to ST-J1 is unclear. In the case of other frameshift mutations causing truncated predicted protein products in NL-2, the potential impact in vitro may be more apparent. For example, in NL-2, ORF16 and ORF138 are both predicted to encode for truncated proteins relative to ST-J1 and other fully sequenced CyHV-2 strains. ORF16 and ORF138 are predicted to encode for type III and type I viral membrane proteins, respectively, both of which are important for herpesvirus viral entry and egress [61,62]. Although potentially important for viral entry and egress, these viral membrane protein truncations in the NL-2 isolate do not totally prevent host cell entry or egress in vitro, but it remains possible that they may reduce fitness relative to ST-J1 in this environment (Figure 6C). However, it should be noted that the same truncated membrane proteins did not prevent NL-2 from exhibiting a much higher virulence and transmission in vivo relative to ST-J1 (Figure 7), although smaller differences elsewhere in either strains may also contribute to these phenotypic observations.

Conversely, other frameshifts in NL-2 relative to ST-J1, such as those in ORF25B and ORF151A, represent truncations of these protein products in ST-J1 relative to NL-2 and other fully sequenced CyHV-2 strains. Notably, ORF25B is a type I viral membrane protein and may therefore be important for viral entry and egress. However, this ORF25B truncation in ST-J1 does not reduce its fitness in vitro relative to NL-2 (Figure 6C). While we observe a lack of virulence and transmission when ST-J1 is delivered by immersion in vivo (Figure 7), it is unclear if this is directly connected to this ORF25B truncation. Notably, in CyHV-3, we previously provided evidence that mutations in type I membrane proteins can impact virion stability in extracellular environments such as mucosal surfaces [59], and it would be interesting to investigate this possibility with CyHV-2 ORF25B in the future in a similar manner. In contrast to other major gene disruptions between NL-2 and ST-J1, the in vivo impact of the truncation of ORF151A in ST-J1 may be more readily apparent. ORF151A encodes for a protein that is predicted to be a viral receptor for Tumor Necrosis Factor (TNF) and is a member of the TNF receptor (TNFR) family of proteins that are encoded by CyHV-1, CyHV-2, and CyHV-3 [60]. TNF is an important cytokine of the innate immune response and is produced in fish in the early stages of viral infection in response to the nuclear factor-κB (NF-κB) inflammatory pathway. Through interaction with its natural cellular receptors (cellular TNFRs), TNF stimulates signaling that leads to an inflammatory response in order to help recruitment of fish immune cells to the site of infection, stimulate their activation, and also cause behavioral fever [63,64,65,66], all of which are important for rapid clearance of viral infections. However, virally encoded TNFRs can act as “decoy” TNFRs, preventing interaction with the cellular TNFRs and thus reducing the antiviral effects of cellular TNF [63,66]. Indeed, we previously demonstrated that a soluble decoy TNFR expressed by CyHV-3 can interfere with behavioral fever response during infection [67]. However, ST-J1 encodes a truncated form of ORF151A (129AA) in which the TNFR domain is missing. Conversely, unlike ST-J1, NL-2 and other CyHV-2 strains encode a full-length protein that includes the TNFR domain (324 AA) (InterProScan analysis, data not shown). Therefore, it is possible that the ability of the ST-J1 strain to counteract host cell TNF during infection is compromised due to this missing decoy TNF receptor encoded by the full-length ORF151A. Given the roles that TNF plays during viral infection, this deficiency may have a greater impact in vivo than in vitro, leading for ST-J1 to exhibit an attenuated phenotype in vivo, relative to NL-2. This may partially account for the opposite patterns we observed between ST-J1 and NL-2 in terms of in vitro and in vivo fitness. Notably, ST-J1 is the only fully sequenced CyHV-2 strain that has been observed to encode truncations in both ORF25B and ORF151A, and it is possible that the reduced virulence in vivo relative to that of NL-2 may be caused by the combined impact of both these frameshifts. Taken together, the genomic and biological comparisons of CyHV-2 from this study have allowed the formation of some interesting hypotheses on some potential determinants of CyHV-2 virulence in vivo, which provide scope for further investigation in future studies.

According to the literature, laboratory challenges with CyHV-2 are primarily conducted via intraperitoneal (IP) injection of infected tissue homogenates or cell culture fluid containing the virus, which can rapidly induce clinical symptoms and high mortality [56,68,69,70,71]. However, this highly artificial challenge method has significant drawbacks. It amplifies the pathogenicity and virulence of the virus, fails to represent the series of events that occur during a natural infection, and does not reflect the natural immune response of the host during a CyHV-2 infection. In fish, mucosal surfaces, including the skin, gills, and gastrointestinal mucosa, serve as the primary entry routes for viruses [72], and previously, we identified the skin as a major entry portal for CyHV-2 [15]. Therefore, identifying an in vivo infection model that will allow us to simulate this natural infection process via the mucosal surfaces is crucial in order to have biologically relevant infection experiment designs. In this study, the inoculation of adult goldfish with NL-2 by immersion using a final concentration of 2000 PFU/mL led to 40% mortality in goldfish (Figure 7). Notably, this dose is 50 times lower than the dose we previously used in equivalent experiments with the YC-01 strain (1 × 10^5^ PFU/mL) [15], which, despite the higher dose, led to little or no mortality in similar adult Shubunkin goldfish. This would indicate that the NL-2 Shubunkin goldfish model is ideal for future studies of CyHV-2, maximizing the biological relevance of observations, whereby it also represents a useful tool to the wider CyHV-2 research community.

## 5. Conclusions

Three isolates of the Dutch CyHV-2 isolates from goldfish or wild gibel carp, NL-1, NL-2, and NL-3, were characterized in this study. They were found to be genetically distinct from the ST-J1 CyHV-2 reference strain and from each other. They were all found to cause disease associated with CyHV-2 in experiments that involved infection of adult Shubunkin goldfish by either IP injection or cohabitation. Among these, NL-2 could be stably passaged in vitro, and it also exhibited the most efficient replication in vitro. NL-2 was also capable of inducing CyHV-2-related mortality in adult Shubunkin goldfish through inoculation by immersion. These two properties make the NL-2 Shubunkin model an optimum in vivo infection model to use in future studies, as it facilitates both stable maintenance and generation of a viral stock in vitro and the infection of hosts in a way that is more representative of a natural infection with CyHV-2. Importantly, the genetic and phenotypic characterization performed in this study generates hypotheses on the potential roles of CyHV-2 genes in adaptation of the virus in vitro or in vivo.

## Figures and Tables

**Table 1:** Summary of genomic variants identified in each of the newly sequenced isolates relative to the CyHV-2 ST-J1 reference strain. Variant classes are defined as per https://www.ensembl.org/info/genome/variation/prediction/classification.html (accessed on 1 April 2025). Consequence types are as defined by the Sequence Ontology (SO) project and are summarized here: https://www.ensembl.org/info/genome/variation/prediction/predicted_data.html (accessed on 1 April 2025).

		NL-1		NL-2		NL-3	
		Number	Proportion	Number	Proportion	Number	Proportion
Variants Class	Indel	13	2.2%	27	3.7%	23	3.1%
	Substitution	40	6.6%	45	6.1%	31	4.2%
	Insertion	117	19.4%	130	17.7%	153	20.5%
	Deletion	138	22.9%	179	24.4%	165	22.1%
	SNV	295	48.9%	353	48.1%	374	50.1%
Consequences	Stop gained	0	0%	0	0%	1	0.1%
	Frameshift *	5	0.8%	7	1%	8	1.1%
	Inframe insertion	29	4.8%	33	4.5%	41	5.5%
	Inframe deletion	48	8%	61	8.3%	55	7.4%
	Protein altering	2	0.3%	5	0.7%	5	0.7%
	Missenses	93	15.4%	118	16.1%	102	13.7
	Start retained	1	0.2%	0	0%	2	0.3%
	Stop retained	0	0%	1	0.1%	1	0.1%
	Synonymous	106	17.6%	125	17%	124	16.6%
	Coding sequence	1	0.2%	1	0.1%	2	0.3%
	Upstream gene	306	50.7%	358	48.8%	390	52.3%
	Downstream gene	9	1.5%	22	3%	11	1.5%
	Intergenic	3	0.5%	3	0.4%	4	0.5%

* This includes multiple frameshifts in the same ORF(s).

## Data Availability

The data presented in this study are available on request from the corresponding authors.

## Supplementary Materials

The following supporting information can be downloaded at https://www.mdpi.com/article/10.3390/v17050658/s1: Figure S1. Representative examples of autopsy observations in Shubunkin goldfish undergoing mortality after IP infection with p#0 inoculums of novel CyHV-2 isolates. Figure S2. Representative examples of clinical symptoms in Shubunkin goldfish after infection with CyHV-2 NL-2 isolate by immersion. Table S1. Summary of NL-1 variants. Table S2. Summary of NL-2 variants. Table S3. Summary of NL-3 variants. Table S4. Summary of NL-1 variant effects. Table S5. Summary of NL-2 variant effects. Table S6. Summary of NL-3 variant effects.
